# Tolerance analysis of chloroplast *OsCu/Zn-SOD* overexpressing rice under NaCl and NaHCO_3_ stress

**DOI:** 10.1371/journal.pone.0186052

**Published:** 2017-10-11

**Authors:** Qingjie Guan, Xu Liao, Mingliang He, Xiufeng Li, Zhenyu Wang, Haiyan Ma, Song Yu, Shenkui Liu

**Affiliations:** 1 Key Laboratory of Saline-alkali Vegetation Ecology Restoration (SAVER), Ministry of Education, Alkali Soil Natural Environmental Science Center (ASNESC), Northeast Forestry University, Harbin, China; 2 Lab of Soybean Molecular Biology and Molecular Breeding, Northeast Institute of Geography and Agroecology, Chinese Academy of Sciences, Nangang District, Harbin City, Heilongjiang, China; Institute of Genetics and Developmental Biology Chinese Academy of Sciences, CHINA

## Abstract

The 636-bp-long cDNA sequence of *OsCu/Zn-SOD* (AK059841) was cloned from *Oryza sativa* var. Longjing11 via reverse transcription polymerase chain reaction (RT-PCR). The encoded protein comprised of 211 amino acids is highly homologous to Cu/Zn-SOD proteins from tuscacera rice and millet. Quantitative RT-PCR revealed that in rice, the level of OsCu/Zn-SOD gene expression was lowest in roots and was highest in petals and during the S5 leaf stage. Moreover, the expression level of OsCu/Zn-SOD gene expression decreased during the L5 leaf stage to maturity. The level of OsCu/Zn-SOD gene expression, however, was increased under saline–sodic stress and NaHCO_3_ stress. Germination tests under 125, 150, and 175 mM NaCl revealed that *OsCu/Zn-SOD*-overexpressing lines performed better than the non-transgenic (NT) Longjing11 lines in terms of germination rate and height. Subjecting seedlings to NaHCO_3_ and water stress revealed that *OsCu/Zn-SOD-*overexpressing lines performed better than NT in terms of SOD activity, fresh weight, root length, and height. Under simulated NaHCO_3_ stress, *OsCu/Zn-SOD-*overexpressing lines performed better than NT in terms of survival rate (25.19% > 6.67%) and yield traits (average grain weight 20.6 > 18.15 g). This study showed that *OsCu/Zn-SOD* gene overexpression increases the detoxification capacity of reactive oxygen species in *O*. *sativa* and reduces salt-induced oxidative damage. We also revealed the regulatory mechanism of OsCu/Zn-SOD enzyme in saline–sodic stress resistance in *O*. *sativa*. Moreover, we provided an experimental foundation for studying the mechanism of OsCu/Zn-SOD enzymes in the chloroplast.

## Introduction

Plants adapt to various environmental conditions by actively regulating their internal metabolism. The enzyme superoxide dismutase (SOD, *EC 1*.*15*.*1*.*1*) determines the reaction rate in any metabolic pathway in plants. SOD is the most important enzyme that responds to reactive oxygen species (ROS) and is specifically produced by the biological PSI–PSП system. SOD catalyzes the superoxide anion, a free radical, into H_2_O_2_ [1]. Generally, the tolerance to oxidative stress is an important factor that affects lifespan, and the activity of ROS-scavenging enzymes is particularly important [2; 3; 4]. When the metabolic cycle of plant cells is disrupted, the activity of Cu/Zn-SOD enzymes is directly affected and superoxide anion radicals accumulate, thus causing oxidative stress [5]. Meanwhile, SOD is the first enzyme to clear active oxygen-free radicals, SOD activity level is associated with oxidation resistance and lifespan [6]. Hitherto, molecular biology, structural biology, and bioinformatics techniques have been used to investigate the factors that affect SOD activity at the sequence and structure level [7; 8; 9]. Some studies have shown that C_24_H_26_O_5_ enhances SOD activity, which signifying increased plant responses against damage [10]. The addition of exogenous proline and glycine betaine enhances the antioxidant defense system of *Oryza sativa*, the SOD activity of glyoxalase systems, and the resistance to salt-induced oxidative stress [11].

To improve genetic resources, genetic engineering has been performed by scientific workers in various countries and the study of transgenic plants has expanded. The SOD gene has been cloned from many plant species. Transgenic tobacco with the rice (*Oryza sativa*) chloroplast SOD gene has salt, water, and PEG stress tolerance; moreover, the tobacco chloroplast antioxidant system has been optimized [12]. Transgenic wheat protoplasts with the tobacco Mn-SOD gene showed lower levels of oxidative damage and higher levels of SOD and GR activities [13]. Transgenic *Populus* L. that overexpression of the *Tamarix androssowii* Mn-SOD gene has enhanced salt tolerance as a result of SOD activity being increased; furthermore, the relative biomass of the transgenic plants increased by 8- to 23-fold [14]. Put Cu/Zn-SOD-overexpressing *Arabidopsis thaliana* has enhanced resistance to saline–sodic stress and growth tolerance to H_2_O_2_ stress [15]. In Mn-SOD-overexpressing *A*. *thaliana* shows that the tolerance to NaCl stress was increased and the levels of Mn-SOD, Cu/Zn-SOD, Fe-SOD, CAT, and POD expression also have been improved under salt stress [16]. The overexpression of tobacco Mn-SOD/APX in transgenic tall fescue plant confers increased tolerance to MV, H_2_O_2_, Cu, Cd, and as stresses as a result of SOD and APX enzyme activities being increased [17]. The increase of SOD, CAT, GR and APX activities enhances tolerance to sulfur dioxide (SO_2_) stress in transgenic Chinese cabbage plants that overexpress the *A*. *thaliana* Mn-SOD/CAT gene [18]. The overexpression of Mn-SOD (*Nicotiana plumbaginifolia*) + Fe-SOD (*A*. *thaliana*) in transgenic alfalfa enhances resistance against drought stress and improves photosynthetic efficiency [19]. The overexpression of cytosolic copper/zinc superoxide dismutase (Cu/Zn-SOD) from the mangrove plant *Avicennia marina* in indica rice var Pusa Basmati enhances tolerance to oxidative, salt, and drought stresses [20]. Saline-sodic stress is caused by salt and pH variation. The in vivo accumulation of ROS injures cell membranes via lipid oxidation, and the accumulation of a large amount of membrane lipid peroxidation products, such as MDA is a cause or product of membrane lipid peroxidation, and also is one of the most obvious indicators of oxidative stress [21; 22]. So far, there have been few reports on the regulatory effects of CuZn-SOD overexpression on saline–sodic stress tolerance in rice.

In this study, we cloned the chloroplast OsCu/Zn-SOD gene in *O*.*sativa* Long jing11, which belongs to one of the eight SOD genes identified in rice including 5 Cu/ZnSODs, 2 FeSODs, and 1 MnSOD[23]. We performed subcellular localization analysis to verify that OsCu/Zn-SOD localized in the chloroplast. Then, we compared the resistance of transgenic rice to NaCl stress with that of non-transgenic (NT) rice during germination and the tolerance of transgenic rice to sodic salt (NaHCO_3_) stress with that of NT rice during the seedling stage of growth. We compared the physiological indexes of OsCu/Zn-SOD-overexpressing transgenic with NT rice lines under NaHCO_3_ stress during vegetative growth. We simulated the effects of OsCu/Zn-SOD gene overexpression on seed-setting in rice under sodic salt stress. Our study provides the experimental foundation for analyzing the protective mechanism of chloroplast Cu/Zn-SOD in salt–sodic-induced oxidative stress and for novel rice cultivars with tolerance for saline–sodic stress.

## Materials and methods

### Plant material

Seeds of *O*. *sativa* L. var. Longjing11 were maintained at Northeast Agricultural University. We selected a white *Allium cepa* variety and an *A*. *thaliana* ecotype. Columbia seeds were maintained and propagated in sodic soil at the Natural Environmental Research Center of Northeast Forestry University.

### Strains and vectors

*Escherichia coli* JM109 and *Agrobacterium tumefaciens* EHA105 were maintained in our laboratory. The pMD18-T vector was procured from TaKaRa Company. The pCXSN and pBI121*-MCS-GFP* botanical expression vectors were maintained saved in our laboratory.

### Reagent

Various restriction enzymes, T_4_-DNA ligase, and gel recovery reagents were obtained from Fermentas. Ex-Taq DNA polymerase and reverse transcription kits were purchased from Invitrogen. Real-time quantitative fluorescent dyes and SYBR Green QPCR Master Mix were purchased from Toyobo (Japan). Northern blot kits were purchased from Roche. Antibiotics were purchased from Sigma-Aldrich. All other analytical reagents were made in China.

### Cloning of chloroplast Cu/Zn-SOD gene of rice and vector construction

Premier 5.0 software was used to design RT-PCR primers based on the nucleotide sequence for *Cu/Zn-SOD* of rice (AK059841, LOC_Os08g44770 *OsCu/Zn-SOD*,) that were retrieved from the GenBank gene registry. Takara Ex-Taq high fidelity enzyme was used to synthesize the following primer sequences: *OsCu/Zn-SOD*-P1 F1: 5’-tatcatcgtcaggtcaggca-3’; *OsCu/Zn-SOD*-R1: 5’-acacttcagctgcaacttgc-3’. Total RNA was extracted from seedling leaves (four leaves and a bud) for the reverse transcription of the cDNA template. The *OsCu/Zn-SOD* gene fragment was amplified and cloned via PCR.

PCR conditions consisted of pre-denaturation at 95°C for 5min; followed by 35 cycles of: 95°C for 30 s, 55°C for 30 s, 72°C for 45s, and and an extension of 72°C for 10 min. The PCR products were resolved by 1% agarose gel electrophoresis. After the target DNA fragment was then retrieved, they were linked with pMD18-T vector. Subsequently, they were transformed to JM109, and then sent to Beijing Genomics Institute (BGI) for restriction enzyme digestion.

To identify the subcellular localization of *OsCu/Zn-SOD* protein, Kpn I and Spe I were added to the 5′ and 3′ ends, respectively, of the *OsCu/Zn-SOD* open reading frame (ORF). The primer OsCu/Zn-SOD-P2 was then designed with the following sequences: F2: 5′-cttggtacc atgcaagccatcctcgcc-3′; *OsCu/Zn-SOD*-R2: 5′-catactagtcaacggggtcagcccaa caac-3′. The primers were synthesized by Shanghai Biological Company. The 1% plasmid of pMD18-*T*::*OsCu/Zn-SOD* was used as the PCR template. The pBI121-*OsCu/Zn-SOD*::*GFP* expression vector was constructed via enzyme digestion, ligation, and transformation.

To construct the overexpressing vector initiated by the 35S promoter, we amplified *OsCu/Zn-SOD* gene via PCR with the following primers: *OsCu/Zn-SOD* P3 F2: 5′-cttggtaccatgcaagccatcctcgcc-3′; *OsCu/Zn-SOD*-R1: 5′-acacttcagctgcaac ttgc-3′. The products were then ligated and then transformed into *pCXSN-T*. After restriction digestion with Xcm I., it shows the identification and construction of *pCXSN*::*OsCu/Zn-SOD* 2D plant expression vector. The vector was then transformed into *Agrobacterium* EHA105 via electric shock transformation. PCR was used to detect successful transformants.

### Quantitative RT-PCR analysis of the tissue-specific expression of OsCu/Zn-SOD gene and expression characteristics under saline alkali stress

Tissue samples were collected from Longjing 11 plants that were pot-cultured in a greenhouse. The tissue samples were frozen in liquid nitrogen at −80°C. To simulate salt and sodic stress, plants were cultured in Hough nutrient solution with 150 mM NaCl and 60 mM NaHCO_3_ for 2, 4, 8, 16, and 24 h. Water was used as the control treatment (0 h stress time). Three leaves and a bud were harvested from each plant. Roots and leaves were also collected and frozen in liquid nitrogen at −80°C.

Total RNA was extracted via Trizol one-step method. First, 1 μg of total RNA template was reverse-transcribed into cDNA and then diluted ten-fold for quantitative PCR. *OsCu/Zn-SOD* gene transcripts were identified via quantitative RT-PCR (qRT-PCR) with the following specific primers for *OsCu/Zn-SOD*-RT-P4: qRT-F: 5′-tatcatcgtcaggtcaggca-3′; qRT-R: 5′-acacttcagctgcaacttgc-3′, Amplification was conducted with the following protocol: 95°C for 5min; followed by 40 cycles of: 95°C for 30 s, 55°C for 30 s, and 72°C for 30 s. PCR was conducted with 2× Brilliant III SYBR Green QPCR Master Mix (Agilent). Data were acquired on the MxPro-Mx3000P system. All gene expression levels were normalized to that of the internal reference actin1 gene (Actin1-F: 5′-cttcatag gaatggaagct gcgggta-3′; Actin1-R: 5′-cgacca ccttgatcttcatgctgcta-3′). No template control (NTC) was set at the same time. Each reaction was repeated thrice. After PCR, relative gene expression was calculated by the system based on the relative quantitative method with the following formula: Rel Exp = 2^−ΔΔCt^, where ΔCt = Ct(*OsCu/Zn-SOD*) − Ct(Actin1); ΔΔCt = (Under stress treatment ΔCt) − (Not under treatment ΔCt). A chart was generated based on fold changes in expression. Excel format was used for the output.

### Detection of the subcellular localization of OsCu/Zn-SOD

pBI121-*OsCu/Zn-SOD*::*GFP* and the chloroplast marker mcherry were transformed into epidermal cells of onion via particle bombardment [24]. Then the onion epidermal cells were placed onto a tablet and the transformants were incubated for 18–24 h. The GFP fluorescence was observed under a confocal microscope (Olympus).

*A*. *thaliana* inflorescence was transformed via infection with *Agrobacterium* EHA105 containing pBI121-*OsCu/Zn-SOD*::*GFP* plasmid DNA using 50 mg/L of T0 Kana screen seed. The T1 generation of Kana-resistant positive lines was detected via PCR (*OsCu/Zn-SOD*-F2/R2) using Tape-Arabidopsis Sandwich extract *A*. *thaliana* leaf T1 protoplast cells [25]. The GFP fluorescence was observed under a confocal microscope (Olympus).

### Tolerance analysis of OsCu/Zn-SOD-overexpressing transgenic rice

Longjing 11 was genetically transformed with the *35S*::*OsCu/Zn-SOD* construct. After rice Longjing 11 seeds were sterilized, 2 mg/L 2, 4-dichlorophenoxyacetic acid was added to the culture medium for callus induction. The calluses were cultured in an illuminated incubator at 30°C for 3 weeks. Naturally split embryogenic calluses were screened on a clean workbench with tweezers. The selected calluses were then co-cultured after 1 week. *Agrobacterium* was treated with electroporation. Then, *pCXSN*::*OsCu/Zn-SOD* plasmid DNA monoclone was placed into 4 ml YEP culture medium with 50 mg/L Kana and 0 mg/L Hyg and then shaking culture at 28°C and 250 rpm for approximately 20–36 h until reaching an OD_λ = 600_ of approximately 0.5. The culture supplemented with acetosyringone (AS, its final concentration is 100 m mol/L) were used to infect the calluses [26, 27].

After screening, differentiation, rooting, and germination with Hyg, T0 generation transgenic plants were obtained and T1 seeds were harvested. After the seeds from NT and T1-#1, #2, #3, #4, and #5 transgenic lines being screened, they were used for Germination tests. Seeds were disinfected with 3% H_2_O_2_ for 30 min and then washed with distilled water. Germination was accelerated by treating seeds with 50 mg/L Hyg aqueous solution at 30°C. After nine–day germination, seedlings with vigorous radicle and plumule were selected for transplantation. To test primer specificity (F2, 5′-ctt ggtaccatgcaagccatcctcgcc-3′, NOS-R, 5′-atcggggaaattcgctagtg-3′) and the genomic condition of the transgenic lines T1-#1, #2, #3, #4, #5, genomic DNA was extracted from the leaves of transgenic *Cu/Zn-SOD* lines via CTAB method [28].

Total RNA was extracted from transgenic, NT, and T3-#1, #2, #3 lines via Trizol one-step method and the 5 μg of the total sample was used for denaturation at 65°C for 10 min. After separation by the 1.5% formaldehyde-agarose gel and then the sample was transferred to the Hybond-N^+^ cellulose nitrate membrane. RNA was cross-linked to the membrane using ultraviolet irradiation. The membrane was crossed by DIG—*chloroplast precursor OsCu/Zn-SOD* (AK059841) DNA specific probes at 50°C for 12 h using CDP-Star^TM^ reagent (Roche company). Signals were detected using LAS 4000 imaging analyzer [29].

NT and T3-#1, #2, #3 seeds were disinfected with 3% H_2_O_2_ for 30 min. The seeds were first cultivated at 30°C to investigate resistance to 0, 125, 150, 175 mM NaCl stress during germination. Germination rate, fresh weight, plant height, and MDA content were determined after 7 days. MDA content under stress treatment was determined via thiobarbituric acid-reactive-substance assay [30].

The growth characteristics of NT and T3-#1, #2, #3 under NaHCO_3_ stress were detected. Germination was accelerated at 30°C in an incubator for 5 days. The uniform-sized seedlings were transferred to Hough stress treatment solutions with 0, 5, 7.5, 10 m mol/L NaHCO_3_ (each treatment replicated three times). Plants were grown in a culture room at 28°C. The nutrient solution was replaced once every 2 days to its original volume. Growth characteristics, plant height, fresh weight, SOD activity, and chlorophyll changes were investigated after 21 days. NBT staining detected the content of superoxide anion in three leaves from seedlings after stress treatment.

### Growth and developmental characteristics of the OsCu/Zn-SOD- overexpressing rice lines under saline–sodic soils

NT and T3-#1, #2, #3 seeds were planted in pots to simulate saline–sodic stress in a field experiment during spring. Field soil and saline–sodic soil patches were obtained from Songneng. Plain and mixed in a basin at ratios of 1:0, 2:1, and 1:1. Groundwater was used for irrigation. Each pot contained three transgenic or NT plants. Ten pots were used for per treatment. The transplant survival rate after 1 month was investigated and plant characteristics under treatment were detected.

## Results

### *OsCu/Zn-SOD* gene cloning, sequence alignment, and phylogenetic tree construction

**Fig 1:**
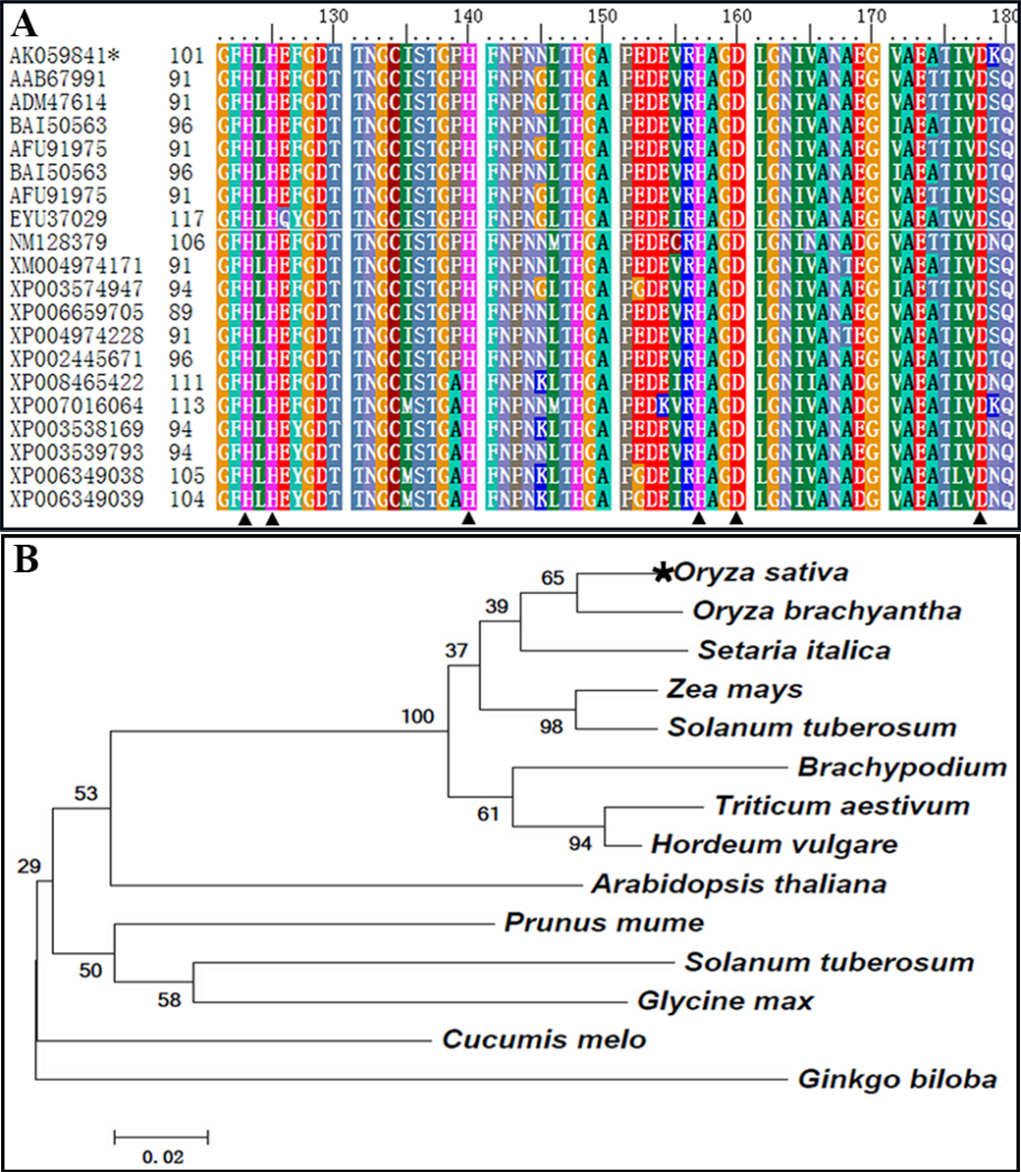
Amino acid sequence alignment and phylogenetic tree of Cu/Zn-SOD proteins from rice and other plant species. (A) Highly homologous regions in the amino acid sequences of *OsCu/Zn-SOD* from different species. The black triangle stands for active site. (B) Phylogenetic tree of *OsCu/Zn-SOD* generated via NJ method.

A 726-bp-long target gene fragment was cloned via RT-PCR. The cloned fragment was then sent to BGI for sequencing via restriction enzyme digestion. As expected, the cloned nucleotide sequence was identical to *OsCu/Zn-SOD* (AK059841). The sequences included an ORF that was 636 bp in length and that encoded 211 amino acids. Sequence alignment revealed that Cu/Zn-SOD protein sequences from different species, such as sorghum, wheat, wild rice, millet, and *A*. *thaliana*, shared 69%–96% homology (Fig 1A). NCBI analysis showed that the sequences contained one Cu^2+^ (His-103,105,120,177) and Zn^2+^ (His-120,128,137,140) binding site and SOD active domain (His-103105120137140177). The cluster analysis and phylogenetic tree of OsCu/Zn-SOD protein and 12 other Cu/Zn-SOD proteins revealed that Cu/Zn-SOD proteins from Longjing 11 rice, wild rice, and millet clustered in the same branch, thus indicating that these three proteins shared the closest evolutionary relationship (Fig 1B).

### qRT-PCR analysis of *OsCu/Zn-SOD* gene expression

**Fig 2:**
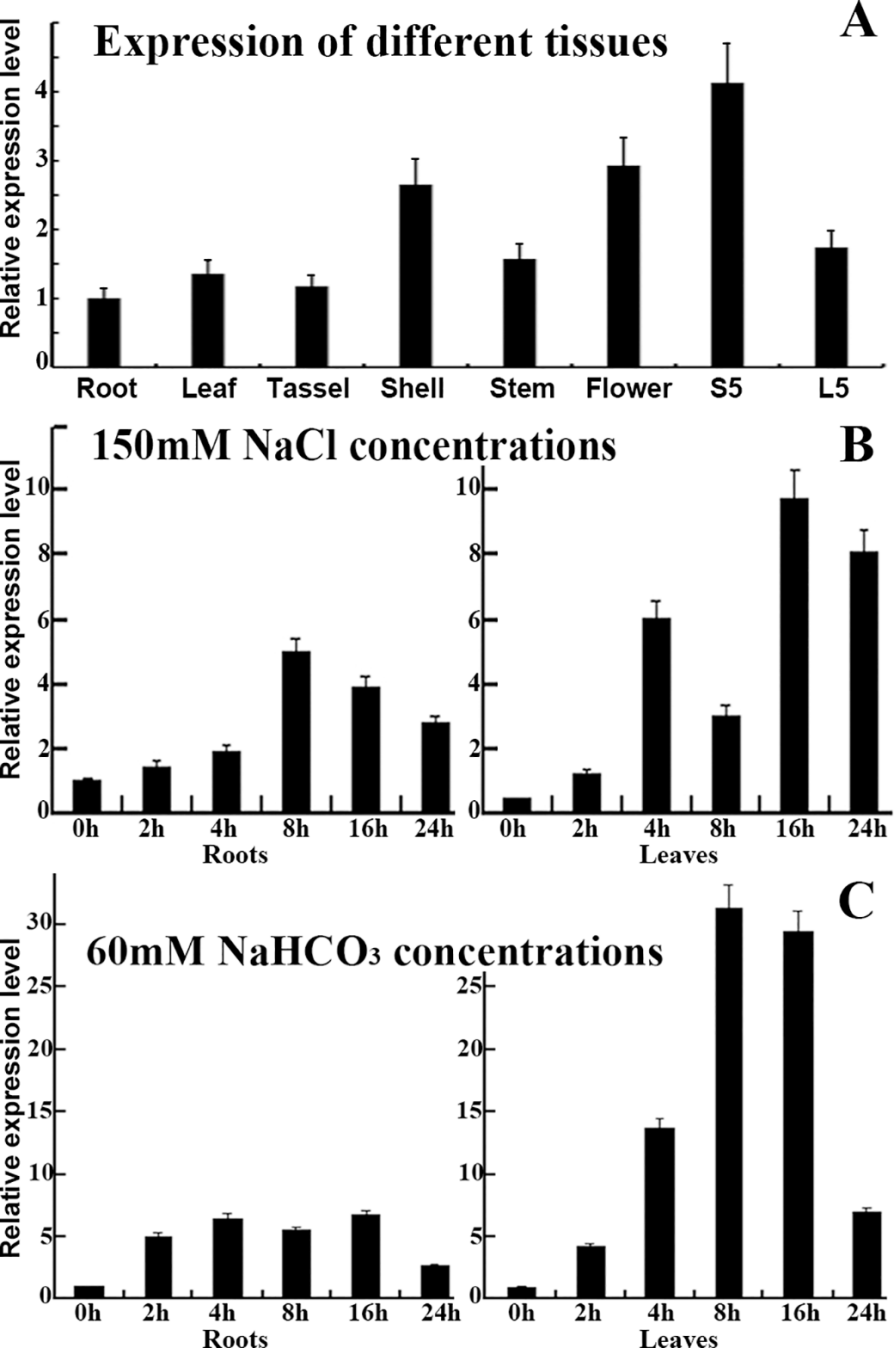
Analysis of *OsCu/Zn-SOD* gene expression. (A) Expression patterns of *OsCu/Zn-SOD* gene in different rice tissues (i.e., root, leaf, tassel, shell, stem, flower, S5, and L5). (B) and (C) *OsCu/Zn-SOD* expression after treatment with 150 mM NaCl or 60 mM NaHCO_3_ for 0, 2, 4, 8, 16 and 24 h.

The specific expression of the *OsCu/Zn-SOD* gene in different rice tissues and organs was analyzed via qRT-PCR. Moreover, the level of transcriptional expression under NaCl and NaHCO_3_ stress was detected. Results showed (Fig 2A) that the level of *OsCu/Zn-SOD* gene expression was in all stages of rice growth and development, and was lowest in roots and highest in flowers and during S5. However, it decreased during the L5 stage of growth. In addition, the level of *OsCu/Zn-SOD* gene expression increased significantly with the increasing duration of exposure to NaCl and NaHCO_3_ stress (Fig 2B and 2C). The level of *OsCu/Zn-SOD* gene expression in leaves was significantly higher than that in the root. Under NaCl stress, the level of *OsCu/Zn-SOD* gene expression increased first and then decreased. The maximum peak expression in roots occurred at 8 h of stress. The maximum peak expression in leaves occurred at 16 h of stress. The relative expression under stress was 9.797-fold higher than that in the control. As a whole, the level of *OsCu/Zn-SOD* gene expression in leaves increased more significantly than in roots. The level of *OsCu/Zn-SOD* gene expression was significantly induced by NaHCO_3_ stress. The maximum peak expression in leaves occurred at 8 h of NaHCO_3_ stress and was 31.362-fold that of the control. The results showed the level of *OsCu/Zn-SOD* gene expression was significantly higher under NaHCO_3_ stress than under NaCl stress. ROS were produced during salt–alkali-induced oxidative stress. We speculated that *OsCu/Zn-SOD* plays a major role in improving the growth of rice when it is subjected to carbonate stress.

### Subcellular localization of OsCu/Zn-SOD

**Fig 3:**
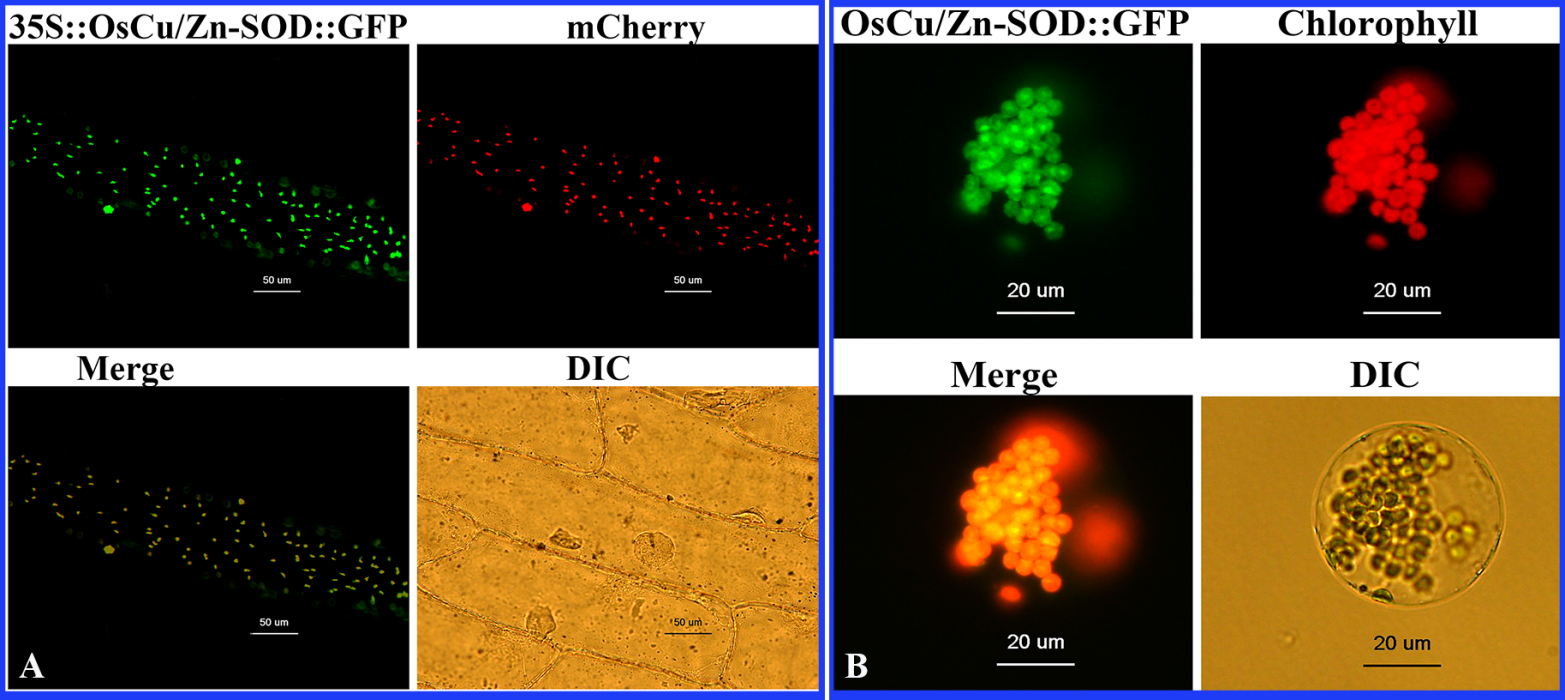
Subcellular localization of the 35S::OsCu/Zn-SOD::GFP fusion protein. (A) To interpret the color references in this figure legend, the reader should refer to the web version of this article. Fluorescence of OsCu/Zn-SOD-GFP fusion protein in onion; merged image of the subcellular localization of the chloroplast marker mcherry; superposition of green fluorescence and mCherry; DIC, bright field. (B) Targeting of GFP fusion proteins to *Arabidopsis* protoplast. Fluorescence of OsCu/Zn-SOD-GFP fusion protein in a protoplast derived from an *Arabidopsis* mesophyll cell; autofluorescence of chlorophyll; merged images from green and red channels; protoplast viewed under white light.

Onion epidermal cells were co-transformed via particle bombardment. The levels of OsCu/Zn-SOD::GFP fusion green fluorescent protein and mCherry red fluorescent protein expression were observed under confocal microscopy. The subcellular localization of OsCu/Zn-SOD in the chloroplast is shown in Fig 3A. In this image, OsCu/Zn-SOD::GFP fusion protein appears as green fluorescent dots and mcherry appears as red fluorescent dots. PCR detection was used to analyze mesophyll protoplast cells from the T1 generation of *Arabidopsis* that was integrated with OsCu/Zn-SOD::GFP. Cells were observed under confocal microscopy (Fig 3B). The green fluorescent channel displayed the green fluorescence of GFP. The spontaneous red fluorescence of chloroplasts was also detected via scanning microscopy. The green and red fluorescence overlapped, showing the yellow chloroplast. The results showed that the OsCu/Zn-SOD::GFP fusion protein localized in the chloroplast, thus verifying the subcellular location of OsCu/Zn-SOD in the chloroplast.

### Identification of *OsCu/Zn-SOD*-overexpressing rice lines

**Fig 4:**
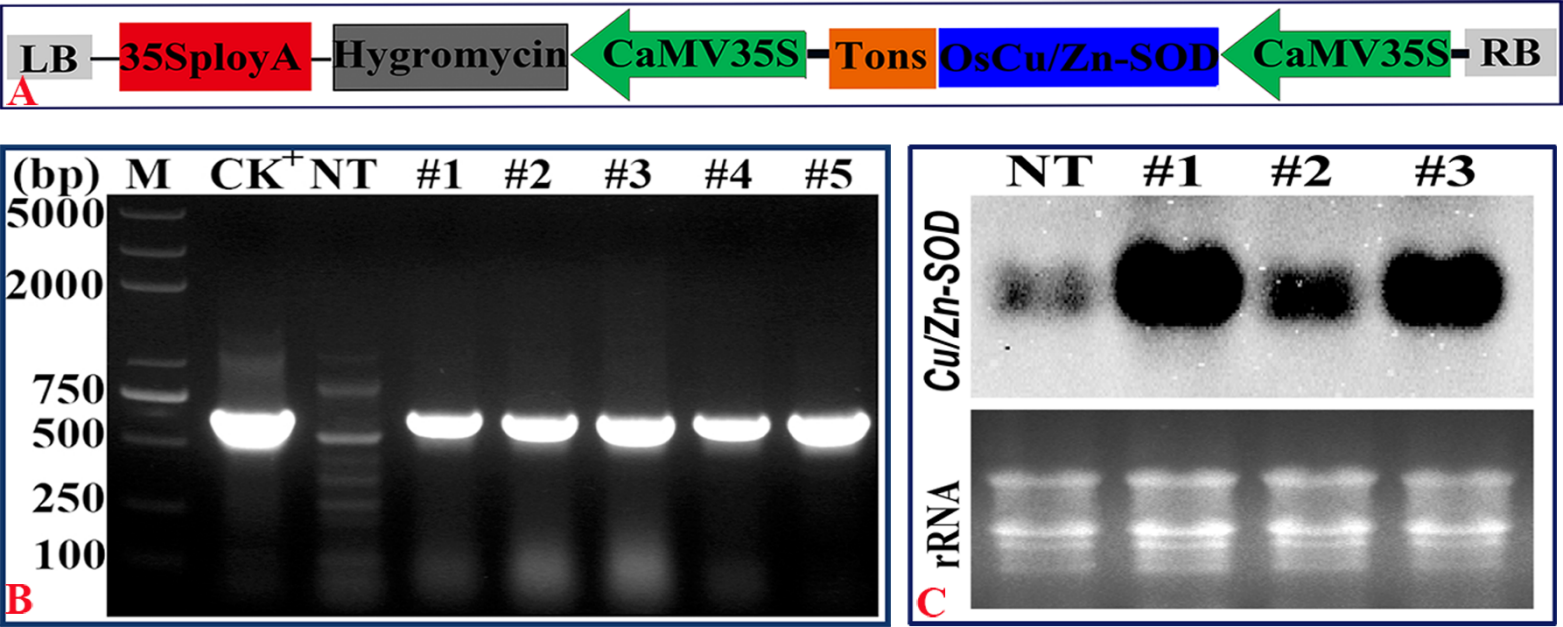
Schematic of the expression vectors pCXSN::*Cu/Zn-SOD* and PCR detection of products. (A). Schematic of the rice *Cu/Zn-SOD* gene two-plant expression vector. The vector starts with CaMV35S and is terminated by Tons. The *PCXSN*::*OsCu/Zn-SOD* expression vector contains the Hyg resistance gene. (B) PCR detection of the CaMV35S::OsCu/Zn-SOD vector in transgenic Longjing 11, generation T1. CK^+^- positive plasmid control; NT- nontransgenic Longjing 11. Lanes 1–5 contain PCR products from *OsCu/Zn-SOD*-overexpressing transgenic Longjing 11 T1 lines. (C) mRNA expression in *OsCu/Zn-SOD-*overexpressing lines, T3 generation (lanes 1–3) as detected by Northern blot.

*pCXSN*::*OsCu/Zn-SOD* plant expression vector was constructed via T-DNA connection method, as shown in Fig 4A. The callus of Longjing 11 were genetically transformed via *Agrobacterium* EHA105-mediated infection. T0-generation transgenic lines were obtained and the T1 generation seeds were then harvested. Germination tests were then conducted in 50 mg/L Hyg solution. Although seeds from both NT and T1-#1, #2, #3 were able to germinate, the plumule color and radicle growth of the lines showed drastic differences after 9 days. The plumule of the transgenic lines was green, whereas that of the NT was white. In addition, transgenic lines showed normal radicle growth (S1), whereas NT showed arrested radicle growth. Unlike NT plants, transgenic Cu/Zn-SOD lines (T1-#1, #2, #3) were able to normally grow, develop, and produce seeds. Moreover, the *OsCu/Zn-SOD*-overexpressing T1-#1, #2, #3, #4, #5 lines are capable of growth in Hyg solution. PCR products were resolved via agarose gel electrophoresis. As shown in Fig 4B, the transgenic lines had 700 bp bands, which is the same as the template plasmid DNA. This result indicated that the *35S*::*OsCu/Zn-SOD* gene was integrated into the genome of the transgenic lines. Northern blot detected the expression of *OsCu/Zn-SOD* gene in T3-#1, #2, #3. Fig 4C shows the expression of *OsCu/Zn-SOD* in #1, #2, #3. In this image, the hybrid signal is clear and that the target gene is overexpressed in T3-#1, #2, #3. Therefore, we harvested the seeds from these plants for subsequent experiments.

### Resistance of *OsCu/Zn-SOD*-overexpressing transgenic rice lines

**Fig 5:**
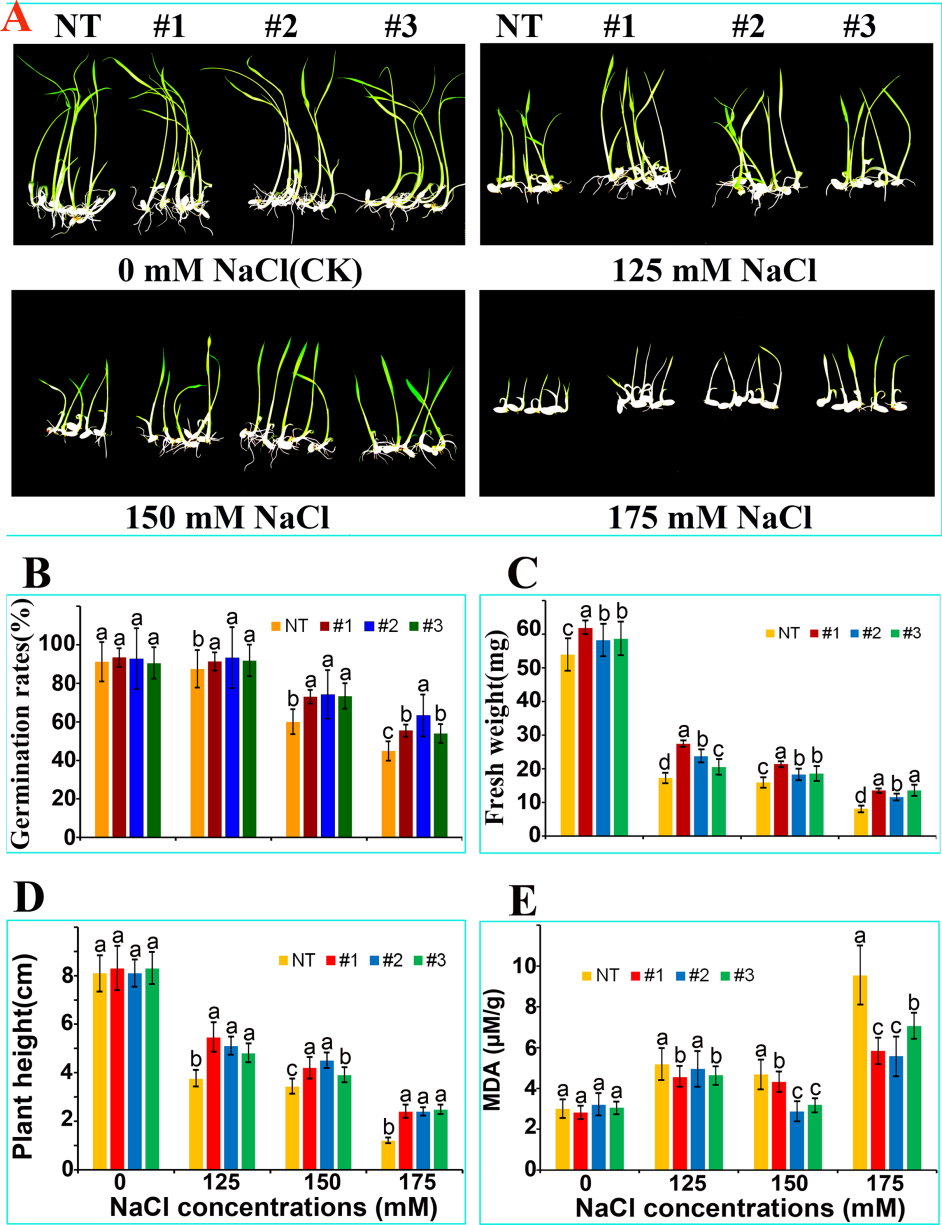
NaCl stress resistance of *OsCu/Zn-SOD*-overexpressing rice lines during germination. (A) Phenotype. (B) Germination rate. (C) Plant fresh weight. (D) Plant height. (E) MDA content of NT and overexpression of *OsCu/Zn-SOD* Rice (T3-#1, #2, #3) after 9 days of germination and growth. Significant differences in parameters when a, b, c, d is p<0.05.

Resistance to NaCl stress during germination NT and *OsCu/Zn-SOD* (T3-#1, #2) lines showed different seed germination rates after 9 days. Although the root and leaf growth of NT and T3-#1, #2 were the same under water treatment (Fig 5A), those of T3-#1, #2, and #3, were superior to that of NT under 125, 150, 175 mM NaCl treatment. Moreover, although germination rate was inhibited under treatment compared with that under the control, the germination rate of the overexpressing lines was higher than that of NT (Fig 5B). In addition, although leaf growth was inhibited by NaCl stress, the overexpressing lines showed better resistance to NaCl treatment and higher fresh weight than NT (Fig 5C). MDA damage to the cell membrane system occurred as a result of salt and oxidative stress. The MDA content in NT was higher than those in the overexpressing lines. (Fig 5D). NBT staining detected that: (1) there was no difference in the color of 5-day rice seedlings of NT and T3 (#1, #2, #3) which germinated under 0mmol/L NaCl stress. (2) Additionally, there was slight difference in color of 5-day rice seedlings between NT and T3 (#1, #2, #3) which germinated under 125mmol/L NaCl stress. (3) Especially, there was a big difference in color of 5-day rice seedlings between NT and T3 (#1, #2, #3) which germinated under 150mmol/L and 175 mmol/L NaCl stress, in mesocotyl (S2). This result indicated that OsCu/Zn-SOD gene overexpression decreases oxidative stress damage and the T3 #1, #2, #3 lines had a certain tolerance to salt stress during germination stage, which were validated by the SOD scavenging activity of these lines.

### Growth characteristics of seedlings under NaCHO_3_ stress

**Fig 6:**
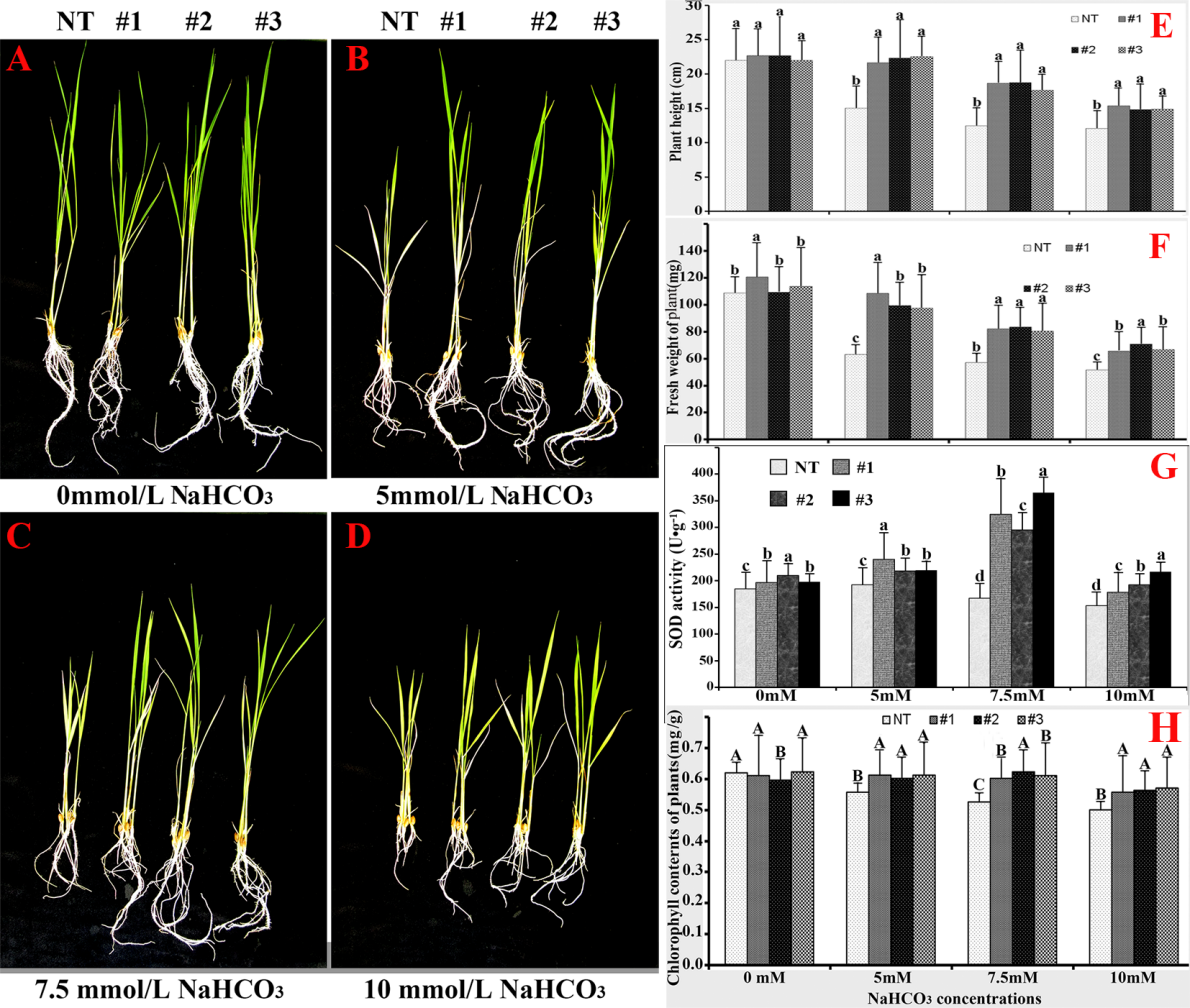
Growth characteristics of *OsCu/Zn-SOD*-overexpressing lines treated with NaHCO_3_. Phenotype A after 21 days of treatment with (A) 0 mM, (B) 5 mM, (C) 7.5 mM, (D) 10 mM NaHCO_3_. (E) Plant height. (F) Fresh weight. (G) SOD activity. (H) Chorophyll content.

To detect the effect of *OsCu/Zn-SOD* gene overexpression on the growth characteristics of rice, we investigated the effect of NaHCO_3_ stress solution on seedlings at 7 d and 21 d after germination. As NaHCO_3_ concentration increased, the growth of NT and T3-#1, #2, #3 lines gradually increased. Although the roots of transgenic lines were much better than those of NT, the leaves of transgenic lines were less green relative to those of NT (Fig 6A, 6B, 6C and 6D). Plant height was not significantly different between NT and transgenic lines. In the stress solution containing 5, 7.5, 10 mM NaHCO3, T3-#1, #2, #3 lines were significantly taller than NT (Fig 6E). The fresh weights per plant of T3-#1, #2, #3 were significantly greater than those of NT (Fig 6F). The SOD activity of transgenic lines was significantly higher than that of NT (Fig 6G). The chlorophyll content of plant leaves from T3-#1, #2, #3 lines (ρ<0.05) were significantly higher than those from NT (Fig 6H). The phenotypic and physiological indicators revealed that the lines of *OsCu/Zn-SOD* gene overexpression were resistant to NaHCO_3_ stress, as well as confirmed that the *OsCu/Zn-SOD* gene increases the resistance of rice to saline–sodic stress during the late stage of germination by increasing ROS-scavenging activity.

**Fig 7:**
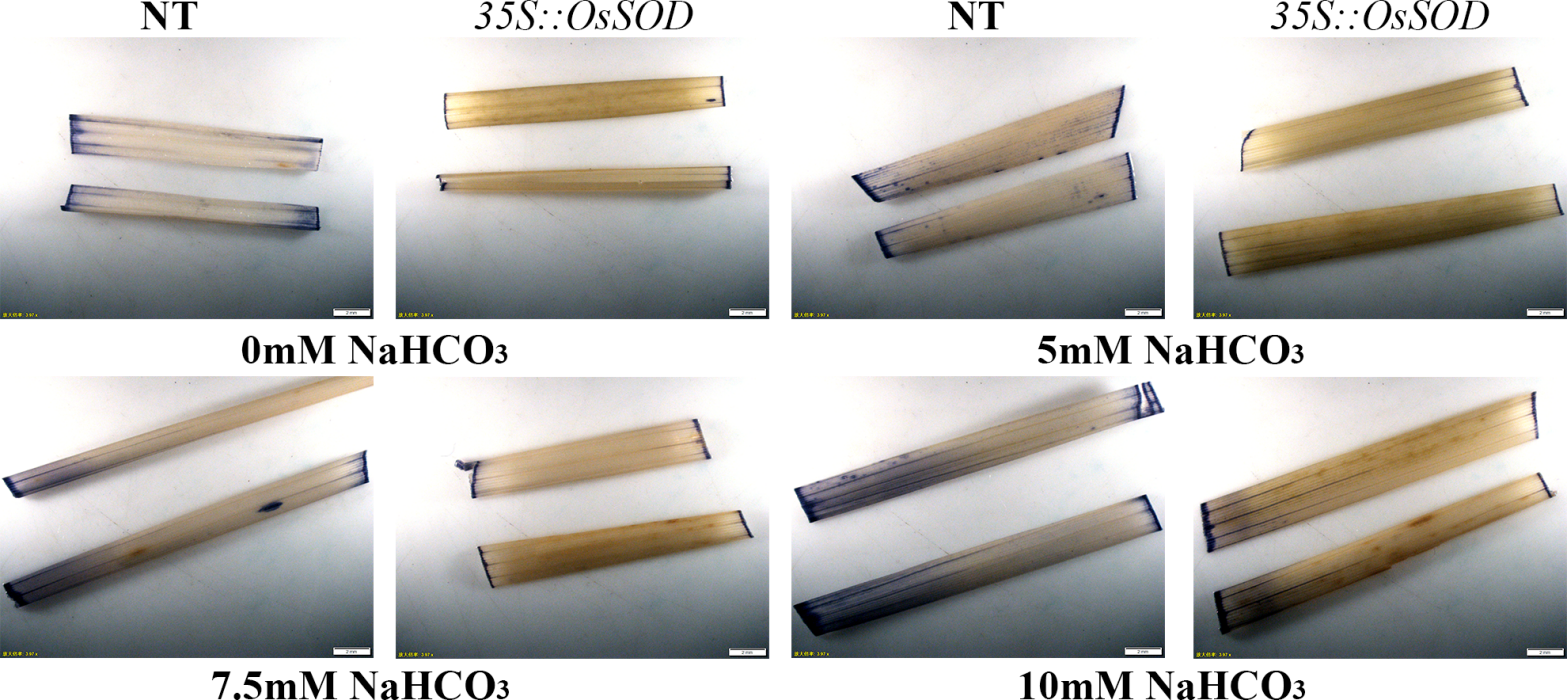
Analysis of active oxygen content in *OsCu/Zn-SOD*-overexpressing lines after 21 days of 0 mM, 5 mM, 7.5 mM, 10 mM stress. NT- nontransgenic rice; *35S*::*SOD*- *OsCu/Zn-SOD-*overexpressing rice.

To detect the relative concentration of superoxide anion in the leaf tissue of *OsCu/Zn-SOD* -overexpressing lines, we detected the difference in superoxide anion concentration between transgenic and NT via NBT staining method. The color depth of the stain reflects the relative content of superoxide anions (Fig 7). The microscopic observation of the *OsCu/Zn-SOD*- overexpressing lines was different from that of NT: as NaHCO_3_ concentration increased, the blue color of the stain gradually deepened. Therefore, the relative concentration of superoxide anion in *OsCu/Zn-SOD*-overexpressing lines was lower than that in NT. This result indicated that SOD activity significantly was increased via *OsCu/Zn-SOD* overexpression, thus enhancing superoxide anion scavenging. This phenomenon improved the growth of transgenic lines as well as enhanced photosynthesis, which was consistent with the high chlorophyll content under NaHCO_3_ treatment.

### Growth characteristics *OsCu/Zn-SOD-*overexpressing and NT lines under saline–sodic stress in the field

**Fig 8:**
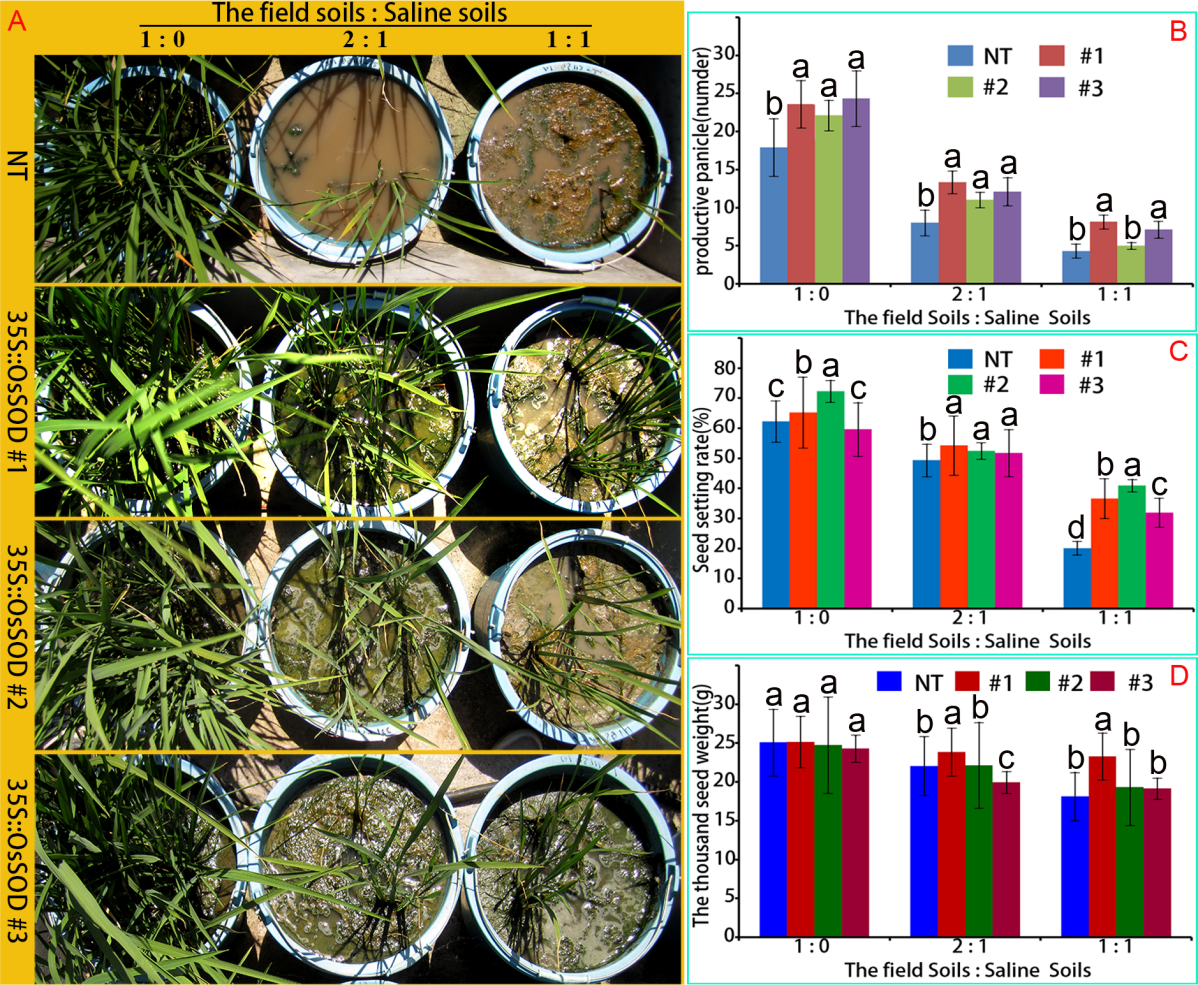
Growth conditions of OsCu/Zn-SOD-overexpressing and NT lines under simulated saline–sodic stress in the field. (A) Phenotype. (B) Productive panicle. (C) Seed-setting rate. (D) Thousand-seed weight.

To simulate rice cultivation in saline–sodic soils during spring, we irrigated transgenic and NT seedlings with groundwater in saline–sodic soil. The different survival characteristics of the two lines after one month (Fig 8). Seedling survival rate was not significantly different (96.667% vs 93.334%) in field soil. However, when grown in field soil with a 2:1 ratio of salinity to sodicity, seedling survival rate was significantly different. The survival rate of *OsCu/Zn-SOD-*overexpressing lines was 73.33%, whereas that of NT was 40%. When grown in field soil with a 1:1 ratio of salinity to sodicity, the seedling survival rate of *OsCu/Zn-SOD-*overexpressing lines was 25.19%, whereas that of NT was 6.67%. Effective tillering rates were investigated in field experiments during autumn. When grown in field soil with a 2:1 ratio of salinity to sodicity, *OsCu/Zn-SOD*-overexpressing lines had 13 tillers/plant and a seed setting rate of 52.5%; NT plants had 8.012 tillers/plant and a seed setting rate of 49.3%. When grown in field soil with a 1:1 ratio of salinity to sodicity, *OsCu/Zn-SOD*-overexpressing lines had more tillers (7.122/plant) than NT (4.318/plant). Furthermore, thousand-seed weight analysis showed that *OsCu/Zn-SOD*-overexpressing lines (average 20.6 g/thousand seed) performed better than NT (average 18.15 g/thousand seed) in terms of yield. Although *OsCu/Zn-SOD*-overexpressing lines were also affected by saline–sodic damage, the lines continued growing and developing, with certain rate of seed setting. These growth responses indicated that the transgenic *OsCu/Zn-SOD* rice has enhanced tolerance to saline–sodic soils.

## Discussion and conclusion

Rice is a staple cereal that is widely consumed all over the world [31; 32; 33]. Regions with saline–sodic soils account for 5% of the earth's arable areas. Salinity–sodicity damage mainly occurs as a result of high Na+ ions and high pH value. Salinity–sodicity damage can slow down the growth and development of plant vegetative organs; decrease the activity of antioxidant enzymes in cells and cause the excessive oxidation of cell membranes, thus leading to metabolic disorders even acceleration of cell senescence and death [34; 35]. The lifespan of organisms is intrinsically associated with the level of SOD enzyme activity and the ability to resist oxidation [36]. However, plants have developed efficient strategies to alleviate the harmful effects of ROS during evolution. The enzymatic system such as superoxide dismutase (SOD) provides a dynamic balance of ROS concentration in plants [37; 38]. The *OsCu/Zn-SOD* gene was cloned from Longjing 11 rice in this study. The cloned *OsCu/Zn-SOD* gene encodes an amino acid sequence that contains Cu^2+^ and Zn^2+^ binding sites and a SOD active domain (Fig 1). qRT—PCR detection showed that saline–sodic stress upregulated the level of *OsCu/Zn-SOD* expression in transcripts, particularly in leaves (Fig 2). This result illustrated that the level of the OsCu/Zn-SOD gene expression in rice during growth and development under salt stress is similar to that of the cytoplasmic copper/zinc superoxide dismutase (Cu/Zn-SOD) gene in the rotifer *Brachionus calyciflorus* under temperature and hydrogen peroxide (H_2_O_2_) stress [39]. The high level of *OsCu/Zn-SOD* expression in leaves is related to its subcellular localization in the chloroplast. The subcellular localization of a protein provides indirect evidence for protein function, which could be further elucidated and verified in future experiments. Protein synthesis in the cytoplasm is guided by a protein screening signal. Proteins may be transported to a specific organelle, secreted outside the cell, or remain in the cytoplasm. Cell function will be profoundly affected if protein localization deviates from the norm [40]. To verify the localization of *OsCu/Zn-SOD* protein in the chloroplastid (Fig 3), we used epidermal cells of onion via particle bombardment and the localization of *OsCu/Zn-SOD* protein in the chloroplastid indicates its function in active oxygen scavenging. Saline–alkali stress induces the excessive production of superoxide anion (O_2_^−^), hydrogen peroxide (H_2_O_2_), and other ROS. What’s worse, under the saline alkali environment, the activity of the antioxidant enzyme protection system was decrased, which causes ROS overproduction and disturbs clearing balance in plants [34; 35; 41; 42]. This study revealed that the *OsCuZn-SOD*-overexpressing lines showed better resistance to NaCl stress during germination than NT (Fig 5). The transgenic lines had a significant advantage over NT in terms of root length, plant height, and fresh weight. These results are similar to those of At *Cu/Zn-SOD*-overexpressing alfalfa lines with enhanced low-temperature resistance [43]. With the increase of membrane lipid peroxidation since the increase in stress intensity, MDA content was increased as increased; therefore, MDA production is an important indicator of the degree of damage to the plant [44]. Although MDA content in transgenic plants and in NT increased after NaCl stress treatment, MDA content in transgenic lines was significantly lower than that in NT (Fig 6), illustrating that the overexpression of *OsCuZn-SOD* gene enhanced ROS scavenging, reduced oxidative stress damage and MDA production. These results are similar to those of overexpressing of the *A*. *marina Cu/Zn-SOD* gene under drought and NaCl stress treatment in rice [45].

Saline–sodic stress is a carbonate-dominated complex salt stress that is characterized by high Na^+^ ions and high pH value. The present study found that the growth of *OsCu/Zn-SOD*-overexpressing lines and NT were all inhibited after NaHCO_3_ stress treatment. Moreover, the degree of damage increased as stress intensity increased (Fig 6); this response is consistent with that under Na_2_CO_3_ treatment [46]. Under 5, 7.5, or 10 mM NaHCO_3_ stress treatment, the *OsCu/Zn-SOD*-overexpressing lines had higher values for height, fresh weight, and chlorophyll index than NT. The Cu/Zn-SOD-overexpressing tobacco plants possess resistance to NaCl, water logging, and PEG stress, as well an optimized chloroplast antioxidant system [47]. Moreover, transgenic tobacco plants that overexpress TaSOD1.1 and TaSOD1.2 have improved physiological functions under NaCl stress, as well as enhanced resistance to salt stress [48]. Under NaCl stress, Cu/Zn-SOD-overexpressing transgenic rice had higher SOD activity than NT, which was consistent with the significant increase in Cu/Zn-SOD activity under the low temperature of 3°C [49]. The overexpression of Cu/Zn-SOD gene also improves excess superoxide radical scavenging and alleviates damage to the plant body. In addition, the content of oxygen free radicals in the leaves of overexpression Cu/Zn-SOD gene rice plants was lower than that in NT (Fig 7). Similarly, the accumulation of active oxygen free radicals decreased when *Kandelia candel*-derived Cu/Zn-SOD is overexpressed in tobacco [50]. The 35S promoter increased the transcription level of the Cu/Zn-SOD gene, thus increasing SOD activity. In turn, the tolerance to NaHCO_3_ stress was increased via the improved ROS balance, which becomes a protective role in chloroplast photosynthesis. When cultivated under field conditions in soils with a 1:1 ratio of salinity to sodicity, the transgenic lines grew and developed better than the NT lines. Therefore, the results of the present study will establish the foundation for the cultivation of rice lines which can be grown on saline–sodic soil.

## Supporting information

S1 Fig Germination tests were then conducted in 50 mg/L Hyg solution. Although seeds from both NT and T1 #1, 2, 3 were able to germinate, the plumule color and radicle growth of the strains showed drastic differences after 9 days.
(DOC)

S2 Fig Effects of the relative concentration of superoxide anion in the 5-day rice seedings of NT and T3 #1, 2, 3, germinated under the NaCl stress. Bars = 2mm.
(DOC)

## Abbreviations

APX: ascorbic acid peroxidase
CAT: catalase
CaMV: Cauliflower mosaic virus
GFP: green fluorescent protein
GR: glutathione reductase
LJ11: LongJing11
MDA: Malondialdehyde
MS: Murashige and Skoog
NT: Non-transgenic rice
POD: peroxidase
ROS: reactive oxygen species
SOD: superoxide dismutase
WT: Wild type

## References

[1] Foyer C. H., & Noctor G. (2003). Redox sensing and signalling associated with reactive oxygen in chloroplasts, peroxisomes and mitochondria. Physiologia Plantarum, 119(3), 355–364.

[2] Martin C. S., Langenbucher J. W., Kaczynski N. A., & Chung T. (1996). Staging in the onset of dsm-iv alcohol symptoms in adolescents: survival/ hazard analyses. Journal of Studies on Alcohol, 57(5), 549–558. 8858553 10.15288/jsa.1996.57.549

[3] Hasty P, Campisi J, Hoeijmakers J, Van Steeg H, Vijg J (2003) Aging and genome maintenance: lessons from the mouse?, Science, 299, 1355–1359. doi: 10.1126/science.1079161 12610296 10.1126/science.1079161

[4] Bubliy O. A., & Loeschcke V. (2005). Correlated responses to selection for stress resistance and longevity in a laboratory population of Drosophila melanogaster Blackwell Science Ltd. 10.1111/j.1420-9101.2005.00928.x 16033550

[5] Asada K. (2006). Production and scavenging of reactive oxygen species in chloroplasts and their functions. Plant Physiology, 141(2), 391 doi: 10.1104/pp.106.082040 16760493 10.1104/pp.106.082040 PMC1475469

[6] Zhu J. K. (2002). Salt and drought stress signal transduction in plants. Annual Review of Plant Biology, 53(53), 247. 10.1146/annurev.arplant.53.091401.143329 PMC3128348 12221975

[7] Ding Y., Cai Y., Han Y., Zhao B., & Zhu L. (2012). Application of principal component analysis to determine the key structural features contributing to iron superoxide dismutase thermostability †. Biopolymers, 97(11), 864–872. doi: 10.1002/bip.22093 22899361 10.1002/bip.22093

[8] Merlino A., Russo K. I., Castellano I., De V. E., Rossi B., & Conte M., et al (2010). Structure and flexibility in cold-adapted iron superoxide dismutases: the case of the enzyme isolated from *Pseudoalteromonas haloplanktis*. Journal of Structural Biology, 172(3), 343–352. doi: 10.1016/j.jsb.2010.08.008 20732427 10.1016/j.jsb.2010.08.008

[9] Pedersen H. L., Willassen N. P., & Leiros I. (2009). The first structure of a cold-adapted superoxide dismutase (SOD): biochemical and structural characterization of iron sod from *Aliivibrio salmonicida*. Acta Crystallographica, 65(2), 84–92. 19193992 10.1107/S1744309109001110 PMC2635881

[10] Sheng L Q., Yan J Z., & Liang W G. (2006). The research progress and application situation of curcumin. Science and technology in western China (4), 14–15. (in Chinese with English abstract. Etc.)

[11] Hasanuzzaman M., Alam M. M., Rahman A., Hasanuzzaman M., Nahar K., & Fujita M. (2014). Exogenous proline and glycine betaine mediated upregulation of antioxidant defense and glyoxalase systems provides better protection against salt-induced oxidative stress in two rice (*Oryza sativa* L.) varieties. Biomed Research International, 2014, 757219–757219. doi: 10.1155/2014/757219 24991566 10.1155/2014/757219 PMC4065706

[12] Badawi G. H., Yamauchi Y., Shimada E., Sasaki R., Kawano N., & Tanaka K., et al (2004). Enhanced tolerance to salt stress and water deficit by overexpressing superoxide dismutase in tobacco (*Nicotiana tabacum*) chloroplasts. Plant Science, 166(4), 919–928.

[13] Melchiorre M., Robert G., Trippi V., Racca R., & Lascano H. R. (2009). Superoxide dismutase and glutathione reductase overexpression in wheat protoplast: photooxidative stress tolerance and changes in cellular redox state. Plant Growth Regulation, 57(1), 57–68.

[14] Wang Y. C., Qu G. Z., Li H. Y., Wu Y. J., Chao W., & Liu G. F., et al (2010). Enhanced salt tolerance of transgenic poplar plants expressing a manganese superoxide dismutase from *Tamarix androssowii*. Molecular Biology Reports, 37(2), 1119–1124. doi: 10.1007/s11033-009-9884-9 19830589 10.1007/s11033-009-9884-9

[15] Wu J., Zhang J., Li X., Xu J., & Wang L. (2016). Identification and characterization of a putcu/zn—sod, gene from *Puccinellia tenuiflora*, (turcz.) scribn. et merr. Plant Growth Regulation, 79(1), 55–64.

[16] Wang Y., Ying Y., Chen J., & Wang X. (2004). *Transgenic arabidopsis*, overexpressing Mn-sod enhanced salt-tolerance. Plant Science, 167(4), 671–677.

[17] Lee S. H., Ahsan N., Lee K. W., Kim D. H., Lee D. G., & Kwak S. S., et al (2007). Simultaneous overexpression of both cuzn superoxide dismutase and ascorbate peroxidase in transgenic tall fescue plants confers increased tolerance to a wide range of abiotic stresses. Journal of Plant Physiology, 164(12), 1626–38. doi: 10.1016/j.jplph.2007.01.003 17360071 10.1016/j.jplph.2007.01.003

[18] Tseng M. J., Liu C. W., & Yiu J. C. (2007). Enhanced tolerance to sulfur dioxide and salt stress of transgenic chinese cabbage plants expressing both superoxide dismutase and catalase in chloroplasts. Plant Physiology and Biochemistry, 45(10–11), 822–833. doi: 10.1016/j.plaphy.2007.07.011 17851086 10.1016/j.plaphy.2007.07.011

[19] Rubio M. C., González E. M., Minchin F. R., Webb K. J., Arrese-Igor C., & Ramos J., et al (2002). Effects of water stress on antioxidant enzymes of leaves and nodules of transgenic alfalfa overexpressing superoxide dismutases. Physiologia Plantarum, 115(4), 531–540. 12121459 10.1034/j.1399-3054.2002.1150407.x

[20] Prashanth S. R., Sadhasivam V., & Parida A. (2008). Over expression of cytosolic copper/zinc superox- ide dismutase from a mangrove plant *Avicennia marina* in indica rice var pusa basmati-1 confers abiotic stress tolerance. Transgenres. Transgenic Research, 17(2), 281–291. 10.1007/s11248-007-9099-6 17541718

[21] Wise R. R., & Naylor A. W. (1987). Chilling-enhanced photooxidation: evidence for the role of singlet oxygen and superoxide in the breakdown of pigments and endogenous antioxidants. Plant Physiology, 83(2), 278–282. 16665236 10.1104/pp.83.2.278 PMC1056348

[22] Lv B. S., Li X. W., Ma H. Y., Sun Y., Wei L. X., & Jiang C. J., et al (2013). Differences in growth and physiology of rice in response to different saline-sodic stress factors. Agronomy Journal, 105(6), 1889.

[23] Nath K., Kumar S., Poudyal R. S., Yang Y. N., Timilsina R., & Yu S. P., et al (2014). Developmental stage-dependent differential gene expression of superoxide dismutase isoenzymes and their localization and physical interaction network in rice (oryza sativa, l.). Genes & Genomics, 36(1), 45–55.

[24] Alinsug M. V., Chen F. F., Luo M., Tai R., Jiang L., & Wu K. (2012). Subcellular localization of class ii hdas in *Arabidopsis thaliana*: nucleocytoplasmic shuttling of hda15 is driven by light. PLOS ONE, 7(2), e30846 doi: 10.1371/journal.pone.0030846 22363501 10.1371/journal.pone.0030846 PMC3281883

[25] Wu F. H., Shen S. C., Lee L. Y., Lee S. H., Chan M. T., & Lin C. S. (2009). Tape-arabidopsis sandwich—a simpler arabidopsis protoplast isolation method. Plant Methods, 5(1), 16. 19930690 10.1186/1746-4811-5-16 PMC2794253

[26] Upadhyaya N. M., Surin B., Ramm K., Gaudron J., Schünmann P. H. D., & Taylor W., et al (2000). *Agrobacterium*-mediated transformation of australian rice cultivars jarrah and amaroo using modified promoters and selectable markers. Functional Plant Biology, 27(3), 201–210.

[27] Toki Seiichi, Hara Naho, Ono Kazuko, Onodera Haruko, Tagiri Akemi, Oka Seibi, Tanaka Hiroshi.(2006) Early infection of scutellum tissue with *Agrobacterium* allows high-speed transformation of rice. The Plant Journal,47(6)969–976. doi: 10.1111/j.1365-313X.2006.02836.x 16961734 10.1111/j.1365-313X.2006.02836.x

[28] Sambrook J., and Russell D.W., eds., Huang P.T., Wang J.X., Zhu H.C., Zhang Z.S., Chen H.P., Fan M, Yu W.Y., and He F.C., trans.(2002) Molecular cloning: a laboratory manual, 3rd, Science Press, Beijing, China, pp.461–509. (in Chinese with English abstract. Etc.)

[29] Sambrook J, Fritsch EF, T M. (1989) Molecular Cloning: A Laboratory Manual, 2nd New York: Cold Spring Harbor Laboratory Press 1566–1573.

[30] Hodges D., Forney C., & Prange R. J. (1999). Improving the thiobarbituric acid-reactive-substances assay for estimating lipid peroxidation in plant tissues containing anthocyanin and other interfering compounds. Planta, 207(4), 604–611. 10.1007/s00425-017-2699-3 28456836

[31] Izawa T., & Shimamoto K. (1996). Becoming a model plant: the importance of rice to plant science. Trends in Plant Science, 1(3), 95–99.

[32] Ohtsubo K., & Nakamura S. (2007). Cultivar identification of rice (*Oryza sativa* L.) by polymerase chain reaction method and its application to processed rice products. Journal of Agricultural & Food Chemistry, 55(4), 1501. 17256960 10.1021/jf062737z

[33] Yang Y., Hu C., & Abuomar M. M. (2012). Conversion of glucose into furans in the presence of alcl3 in an ethanol-water solvent system. Bioresource Technology, 116(7), 190. 22609675 10.1016/j.biortech.2012.03.126

[34] Zhao K F. (2002). Plants to salt stress adaptation. Bulletin of Biology, 37(6), 7–10.

[35] Wang H Z., Lu D Z., Yan M X.,Qian Q., & Huan D N. (2002). Study on the Transformation of Rice (Oryza sat iva L.) with GutD Gene. Bulletin of Science and Technology, 18(6), 441–445. (in Chinese with English abstract. Etc.)

[36] Zhu JK (2002). Salt and drought stress signal transduction in plants. Annual Review Plant Biology, 53, 247–273. 10.1146/annurev.arplant.53.091401.143329 PMC3128348 12221975

[37] Gopavajhula V. R., Chaitanya K. V., Akbar A. K. P., Shaik J. P., Reddy P. N., & Alanazi M. (2013). Modeling and analysis of soybean (glycine max. l) Cu/Zn, Mn and Fe superoxide dismutases. Genetics & Molecular Biology, 36(2), 225–236. 23885205 10.1590/S1415-47572013005000023 PMC3715289

[38] Filiz Ertuğrul, & Tombuloğlu Hüseyin. (2015). Genome-wide distribution of superoxide dismutase (SOD) gene families in sorghum bicolor. Turkish Journal of Biology, 39(1), 49–59.

[39] Yang JH, Dong SM, Jiang QC, Si Q, Liu XZ, Yang JX (2013)Characterization and expression of cytoplasmic copper/zinc superoxide dismutase (CuZnSOD) gene under temperature and hydrogen peroxide (H_2_O_2_) in rotifer Brachionus calyciflorus. Gene, 518:388–396. doi: 10.1016/j.gene.2012.12.101 23313880 10.1016/j.gene.2012.12.101

[40] Devos D., & Valencia A. (2000). Practical limits of function prediction. Proteins-structure Function & Bioinformatics, 41(1), 98–107. 10944397

[41] Allen R. D. (1995). Dissection of oxidative stress tolerance using transgenic plants. Plant Physiology, 107(4), 1049–1054. 12228418 10.1104/pp.107.4.1049 PMC157235

[42] Hussain S., Thomassen L. C., Ferecatu I., Borot M. C., Andreau K., & Martens J. A., et al (2010). Carbon black and titanium dioxide nanoparticles elicit distinct apoptotic pathways in bronchial epithelial cells. Particle and Fibre Toxicology, 7(1), 10. 20398356 10.1186/1743-8977-7-10 PMC2873464

[43] Mckersie B. D., Murnaghan J., Jones K. S., & Bowley S. R. (2000). Iron-superoxide dismutase expression in transgenic alfalfa increases winter survival without a detectable increase in photosynthetic oxidative stress tolerance. Plant Physiology, 122(4), 1427–1438. 10759538 10.1104/pp.122.4.1427 PMC58977

[44] Pan X., Cao Q., & Wang G. (2002). Evaluation of lipid peroxidation for use in selection of cold hardiness cultivars of almond. Acta Ecologica Sinica, 22(11), 1902–1911.

[45] Prashanth S. R., Sadhasivam V., & Parida A. (2008). Over expression of cytosolic copper/zinc superox- ide dismutase from a mangrove plant avicennia marina in indica rice var pusa basmati-1 confers abiotic stress tolerance. transgen res. Transgenic Research, 17(2), 281–291. doi: 10.1007/s11248-007-9099-6 17541718 10.1007/s11248-007-9099-6

[46] Lv B. S., Li X. W., Ma H. Y., Sun Y., Wei L. X., & Jiang C. J., et al (2013). Differences in growth and physiology of rice in response to different saline-sodic stress factors. Agronomy Journal, 105(6), 1889.

[47] Badawi G. H., Yamauchi Y., Shimada E., Sasaki R., Kawano N., & Tanaka K., et al (2004). Enhanced tolerance to salt stress and water deficit by overexpressing superoxide dismutase in tobacco (nicotiana tabacum) chloroplasts. Plant Science, 166(4), 919–928.

[48] Zhang H N., LI X J., LI C D., & Xiao K. (2008). Effects of Overexpression of Wheat Superoxide Dismutase (SOD) Genes on Salt Tolerant Capability in Tobacco. Acta Agronomica Sinica, 34(8), 1403–1408.

[49] Zhang Y., Tan J., Guo Z., Lu S., He S., & Shu W., et al (2009). Increased abscisic acid levels in transgenic tobacco over-expressing 9 cis-epoxycarotenoid dioxygenase influence h2o2 and no production and antioxidant defences. Plant Cell & Environment, 32(5), 509–519. 10.1111/j.1365-3040.2009.01945.x 19183289

[50] Jing X., Hou P., Lu Y., Deng S., Li N., & Zhao R., et al (2015). Overexpression of copper/zinc superoxide dismutase from mangrove kandelia candel in tobacco enhances salinity tolerance by the reduction of reactive oxygen species in chloroplast. Frontiers in Plant Science, 6, 23 doi: 10.3389/fpls.2015.00023 25657655 10.3389/fpls.2015.00023 PMC4302849

