# Supplementary material for: Tolerance analysis of chloroplast OsCu/Zn-SOD overexpressing rice under NaCl and NaHCO3 stress
Source: PLoS One. 2017 Oct 11;12(10):e0186052. doi: 10.1371/journal.pone.0186052 (PMC5636109; doi:10.1371/journal.pone.0186052)
Supplement: S1 Fig — Although seeds from both NT and T1 #1, 2, 3 were able to germinate, the plumule color and radicle growth of the strains showed drastic differences after 9 days. (DOC) [file pone.0186052.s001.doc]

Fig. S1


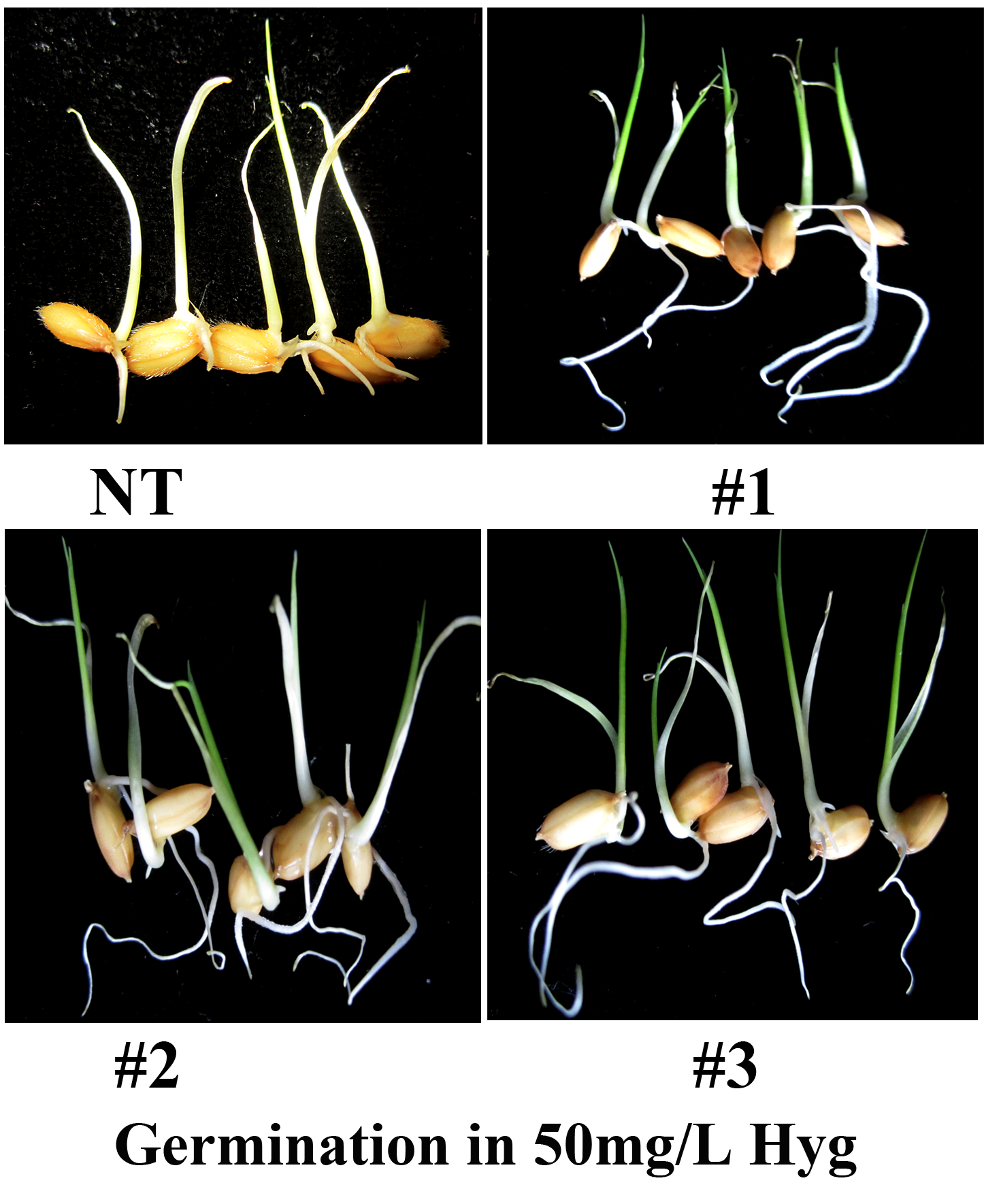


S1. Germination tests were then conducted in 50 mg/L Hyg solution.

Although seeds from both NT and T1-#1, #2, #3 were able to germinate, the plumule color and radicle growth of the strains showed drastic differences after 9 days.
