# Supplementary material for: Tolerance analysis of chloroplast OsCu/Zn-SOD overexpressing rice under NaCl and NaHCO3 stress
Source: PLoS One. 2017 Oct 11;12(10):e0186052. doi: 10.1371/journal.pone.0186052 (PMC5636109; doi:10.1371/journal.pone.0186052)
Supplement: S2 Fig — Bars = 2mm. (DOC) [file pone.0186052.s002.doc]

**Supplementary figure 2：**


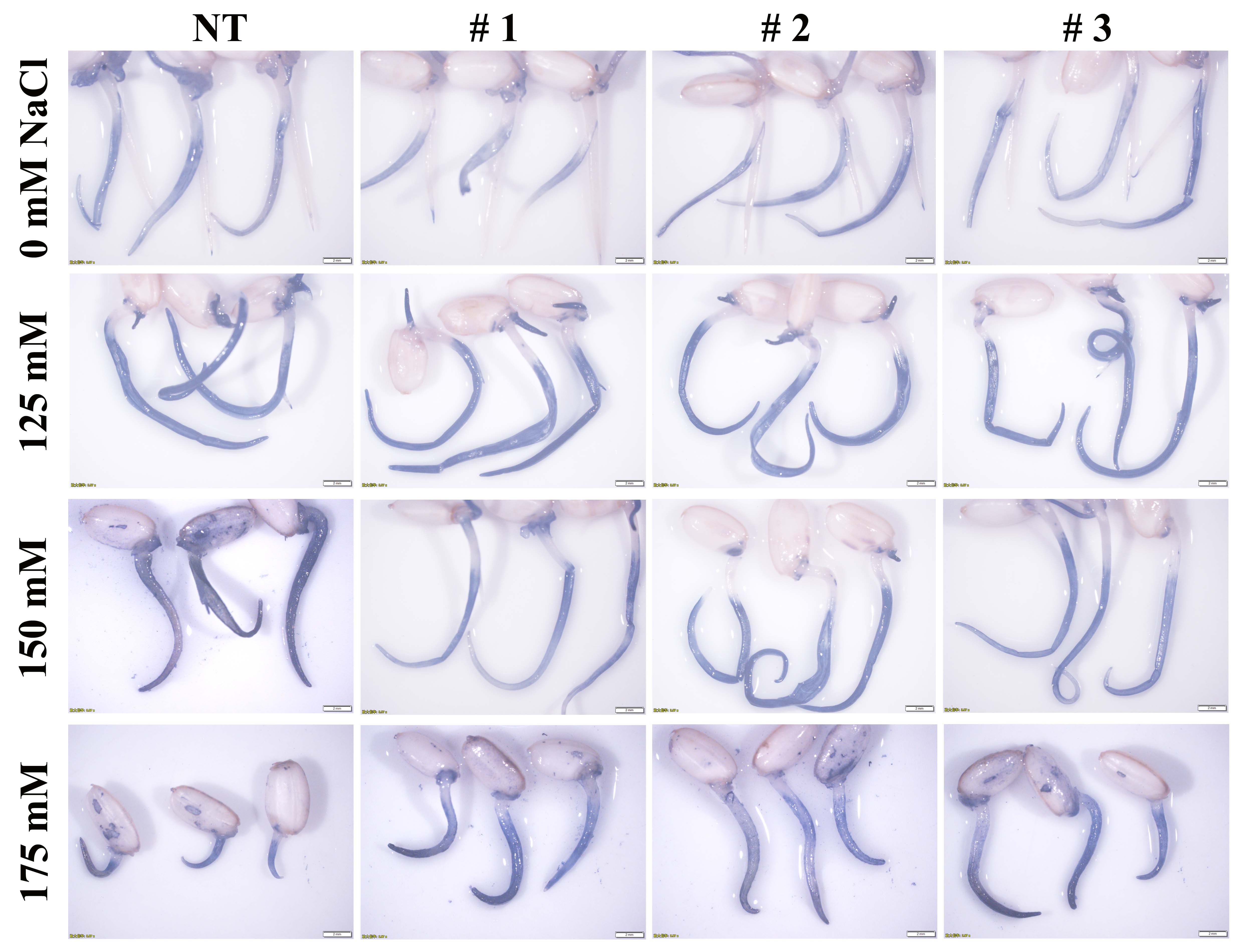


Supplementary figure 2：Effects of the relative concentration of superoxide anion in the 5-day rice seedings of NT and T3 (#1, #2, #3), germinated under the NaCl stress. Bars=2mm.
